# Emerging role of contact-mediated cell communication in tissue development and diseases

**DOI:** 10.1007/s00418-018-1732-3

**Published:** 2018-09-25

**Authors:** Benjamin Mattes, Steffen Scholpp

**Affiliations:** Living Systems Institute, School of Biosciences, College of Life and Environmental Science, University of Exeter, Exeter, EX4 4QD UK

**Keywords:** Cytoneme, Tunnelling nanotubes, Contact-dependent signalling, Paracrine signalling, Wnt, Hedgehog, Trafficking

## Abstract

Cells of multicellular organisms are in continuous conversation with the neighbouring cells. The sender cells signal the receiver cells to influence their behaviour in transport, metabolism, motility, division, and growth. How cells communicate with each other can be categorized by biochemical signalling processes, which can be characterised by the distance between the sender cell and the receiver cell. Existing classifications describe autocrine signals as those where the sender cell is identical to the receiver cell. Complementary to this scenario, paracrine signalling describes signalling between a sender cell and a different receiver cell. Finally, juxtacrine signalling describes the exchange of information between adjacent cells by direct cell contact, whereas endocrine signalling describes the exchange of information, e.g., by hormones between distant cells or even organs through the bloodstream. In the last two decades, however, an unexpected communication mechanism has been identified which uses cell protrusions to exchange chemical signals by direct contact over long distances. These signalling protrusions can deliver signals in both ways, from sender to receiver and vice versa. We are starting to understand the morphology and function of these signalling protrusions in many tissues and this accumulation of findings forces us to revise our view of contact-dependent cell communication. In this review, we will focus on the two main categories of signalling protrusions, cytonemes and tunnelling nanotubes. These signalling protrusions emerge as essential structural components of a vibrant communication network in the development and tissue homeostasis of any multicellular organism.

## An introduction into contact-dependent cell communication

Cell–cell communication by interaction of the receptors and ligands of directly adjacent cells is generally defined as juxtacrine signalling. Here, signalling components bind to their counterparts on the neighbouring cells. Notch–Delta signalling is one of the best-studied examples for such a fundamental communication mechanism that governs the differentiation of many cell types (Fortini 2009). The core Notch signalling pathway contains only a small number of signalling components such as the Notch receptors and its ligands from, for example, the Delta-like and Jagged families. Activation of the Notch receptor by ligand binding triggers its own proteolytic cleavage, leading to subsequent translocation of the intracellular domain of the receptor to the nucleus to initiate the transcription of Notch target genes. Remarkably, both of the main signalling components, the Notch receptors as well as the ligands, are membrane-bound. Initiation of signalling requires, therefore, a close physical interaction of the sender cell with the receiver cell and a precise steric orientation of the transmembrane signalling components to allow interactions between cells (in trans). However, this classical example for juxtacrine signalling process has been called into question. Notch–Delta interaction has also been observed to operate between distant cells in a tissue. How can we solve this contradictory observation compared to the definition of juxtacrine signalling of adjacent cells? An alternative means to localize Notch activation is by positioning Notch signalling components at cellular protrusions, which leads to the activation of signalling at distance (De Joussineau et al. 2003; Cohen et al. 2010). These signalling filopodia can span over several cell diameters and have been defined as cytonemes (Ramírez-Weber and Kornberg 1999). Cytonemes transport a large variety of signalling components in many tissues and organisms (Kornberg and Roy 2014). Our knowledge of cytonemal transport has steadily increased in the last years and we will discuss recent advances in this review.

Another form of contact-dependent and long-range signalling requires the formation of thin membranous, cytoplasmic connections (Gerdes and Carvalho 2008). Through cytoplasmic connections, various types of information can be transmitted. Various experimental settings demonstrate that the biochemical signals—soluble and membrane tethered—can be selectively transported through membranous tubes between cells, which suggest that their membranes and cytoplasm are continuously connected. Due to their structure, these conduits have been termed as tunnelling nanotubes (TNTs). Low molecular weight biochemical signals were not the only components observed in these conduits: vesicles and even organelles enter these tubes on one side, then transport along the tube, and exit into the connected cell (Sisakhtnezhad and Khosravi 2015). During this unidirectional transfer, a continuous and rapid translocation of these structures could be detected at any given point along the conduit, which was consistent with the existence of a direct intercellular transfer mechanism based on membrane continuity. In addition to biochemical signals and organelles, these thin cytoplasm-filled bridges can also be used to transfer electrical and mechanical stimuli from one cell to another. In a following section, we will discuss the function of these TNTs with regard to information exchange.

After examining the recent advances in our understanding of cytonemes and TNTs, we will compare these two kinds of signalling protrusions. We hypothesize that they serve as an underlying structure of an emerging information grid between cells. This information network connects cells with an end-to-end principal for precise collecting, disseminating, and managing information. This is crucial during development of embryonic tissues, for maintaining balance of mature tissues and to facilitate tissue response to a disease in multicellular organism.

## The multiple functions of filopodia

Filopodia are actin-rich membrane protrusions that extend from cells (Mattila and Lappalainen 2008; Jacquemet et al. 2015). These finger-like structures are thin with a diameter of about 100–300 nm. On average, filopodia vary in lengths and reach on average a length of about several micrometres. Very short protrusions emergent from the cell cortex and lamellipodia are often called ‘microspikes’ which can be observed in large numbers. However, in some circumstances filopodia can also extend over several hundreds of micrometres. Filopodia contain parallel-oriented, tight filamentous (F)-actin bundles allowing quick extension and retraction within minutes. Functionally, filopodia are involved in many essential tasks. In general, filopodia have most often been associated with changes in cell shape or in migration of cells and tissues (Ridley et al. 2003). For example, filopodia have been described to influence neurite formation and axon guidance in neurons (Sainath and Gallo 2015). During cell migration, filopodia form initial adhesion sites, which can later be transformed into stable, mature focal adhesions. Finally, tissue migration is another common event during embryonic development and wound healing in which filopodia function is required. Filopodia project at the edges of epithelial cells and have an important role during the movement of these epithelial cell sheets. Cell adhesion molecules allow the ‘tentacles’ to stick to the substrate or to neighbouring cells to promote migration. The presence of filopodia might appear to promote migration; however, there is still an ongoing debate about the level of involvement of filopodia in migratory events.

The dynamic behaviours of filopodia have also suggested an additional sensory role (Heckman and Plummer 2013). The idea is that filopodia act as ‘antenna’ of the cell, to probe their environment. Signals from the environment sensed by filopodia could influence their cell behaviour. Some filopodia contain receptors for a huge variety of signalling molecules and extracellular matrix proteins. For example, a bi-directional signalling interaction of the EphrinB1 ligand on filopodia of hepatic progenitors and the EphB3b receptor on filopodia of cells of the lateral plate mesoderm is important for positioning of the zebrafish liver (Cayuso et al. 2016). As a consequence, filopodia may act as sites for signal transfer. The length and the dynamics of these ‘fishing rods’ make them ideal signal receivers crucial for the development of a tissue. In some circumstances, the signal can also be passed on by filopodia. Filopodia on macrophages have been suggested to relay signals in such a way. In zebrafish, pigment cells project filopodia with signal-containing vesicles at their tips and deposit these in the tissue. These vesicles are taken up by macrophages and subsequently re-distributed to the target cells (Eom and Parichy 2017).

## Cytonemes transmit signalling in Drosophila

**Fig. 1:**
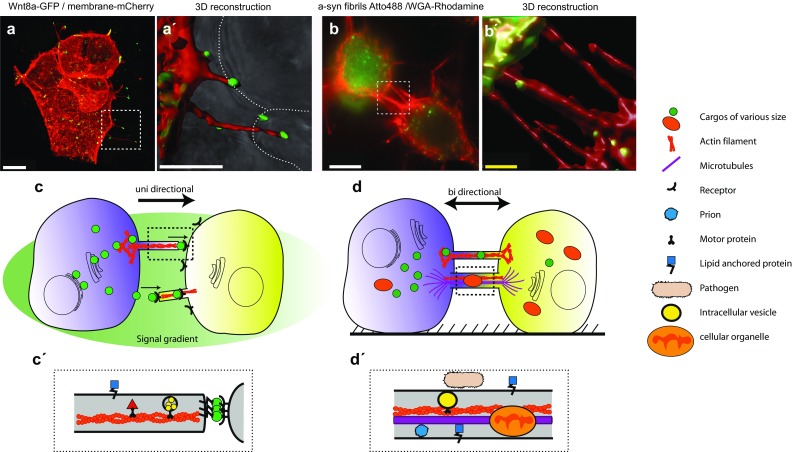
Structure and function of cytonemes and tunnelling nanotubes. **a** Live imaging of Wnt8a-GFP expressing cells and membrane-mCherry expressing zebrafish cells in vivo. Wnt8a-GFP positive cytonemes extend from the source cell to contact unlabelled adjacent cells. **a**’ 3D reconstruction demonstrates the tip localization of the signalling proteins and the interaction with adjacent cells (dotted line). **b** Atto488 α-syn fibrils inside TNTs, labelled with WGA rodamine. (Saida Abounit and Chiara Zurzolo, unpublished figure). **b’** Labelled molecules are transported inside the nanotube. **c** Cytonemes generate a unidirectional signalling gradient by delivering or receiving signalling components. **c**’ Cytonemes consist of thin F-actin bundles and generally rely on ligand–receptor interactions. **d** TNTs can build seamless and stable bi-directional transfer bridges between cells. **d’** Depending on the context and the delivered cargo, TNTs are composed of F-actin in conjunction with microtubules. White scale bars in **a, a’** and **b** 10 µm, yellow scale bar in b’ 1 µm

A special type of long filopodia connected to signalling events had been first noted in Drosophila wing imaginal disc cells by the lab of Thomas Kornberg (Fig. 1c, c’). These protrusions orient uniformly towards the disc midline where the morphogen signalling protein Decapentaplegic (Dpp) is expressed (Ramírez-Weber and Kornberg 1999). The Dpp receptor Thickvein (Tkv) is present in motile puncta in these extensions suggesting that they are used to transport Dpp across the disc (Hsiung et al. 2005). Based on this initial finding, filopodia which are involved in signal distribution because they contain ligands or receptors, have been termed as cytonemes. Cytonemes can also be observed in various other Drosophila tissues; for example, the Egf receptor (EgfR) is present in clusters in the cytonemes that orient to the morphogenetic furrow where the ligand Spi/Egf is expressed (Roy and Kornberg 2011; Peng et al. 2012). Furthermore, the wing disc orchestrates dorsal air sac development by producing Fgf that travel via specific cytonemes to signal to the air sac primordium (Sato and Kornberg 2002). Cytonemes from the myoblasts take up Wingless (Wg) from the imaginal disc (Huang and Kornberg 2015). Recently, signalling protrusions were discovered in the Drosophila male germline stem cell niche to transmit Tkv-dependent Dpp signalling (Inaba et al. 2015). These cytoneme-like protrusions transport signalling molecules in a similar way compared to cytonemes but are microtubule-based and F-actin independent.

In Drosophila, Hedgehog (Hh) trafficking is the best-studied cytoneme-based mechanism so far with numerous contributions from the group of Isabel Guerrero. In both wing disc and abdominal histoblasts, cytonemes from Hh-producing cells extend across its morphogenetic gradient (Bischoff et al. 2013). Additionally, the maintenance of germline stem cells by cytoneme-mediated delivery of Hh has been reported (Rojas-Ríos et al. 2012). The Hh cytonemes extend and retract dynamically and act as conduits for ligand dispersion mainly at the basal plane of the epithelium. Abrogation of these cytonemes affects Hh signalling due to the association of Hh with vesicles which are transported along these cytonemes (Gradilla et al. 2014). These vesicles could be identified as exosomes. Cytonemes similarly emanate from the Hh-ligand-receiving cells (Chen et al. 2017; González-Méndez et al. 2017). Essential Hh signalling components of the receiving cell localize to these cytonemes, including the canonical Hh receptor Patched and the essential co-receptors Smo and Ihog. Formation of the sending and the receiving cytonemes depends on the composition of the extracellular matrix. Cytonemes require heparan sulphate proteoglycans (Bischoff et al. 2013) such as Dally and Dally-like protein for proper extension (González-Méndez et al. 2017; Bilioni et al. 2013). Cytonemes use these proteoglycans to navigate through specific layers of the extracellular matrix (Huang and Kornberg 2016).

There is further evidence that cytonemes form signal-specific platforms. In the Drosophila tracheal air sac primordium, a branch that associates with the wing disc, cytonemes extend from the basal surface of the tracheal epithelium. Some of these cytonemes contain either the Dpp receptor Tkv or the Fgf receptor. So far, cytonemes containing both receptors have not been detected (Roy et al. 2014). This segregation of signal pathway specific receptors to different cytonemes suggests that cells can respond to multiple signals by extending signal-specific cytonemes in invertebrates.

## Cytonemes operate in vertebrate tissue

Recently, there is accumulating evidence that vertebrate cells have a similar ability to form signalling filopodia. In transformed mammalian cell lines, filopodia are associated with the transduction cascade for EGF signalling (Lidke et al. 2005) and FGF signalling (Koizumi et al. 2012). They have been observed extending from B-cells (Gupta and DeFranco 2003) and from mast cells induced by chemokines (Fifadara et al. 2010). Furthermore, cytonemes have also been described regulating the distribution of pigment cells in zebrafish (Hamada et al. 2014). A recent report describes cytonemes as an essential trafficking mechanism for Shh in the chick limb bud (Sanders et al. 2013). The Shh ligand is transported in the anterograde direction in cytonemes with a length of 200 µm. Structurally, Shh cytonemes are characterised by the existence of microtubules at the proximal base. Accordingly, transport of Shh—with a maximum velocity of anterograde particle movement of 120 nm/s—is consistent with actin-based myosin motors. Other cytonemes formed by these chick mesenchymal cells carry the Shh co-receptors Cdo and Boc and connect to the Shh-positive cytonemes. This allows the distribution of the Shh protein over a distance of several hundreds of micrometres in the chick limb bud. In addition to the appearance of cytonemes in the stem cell niche in Drosophila (Inaba et al. 2015), vertebrate stem cells often display a complex web of cytonemal protrusions. Indeed, the intestinal crypt—Lgr4 and Lgr5—have the ability of inducing a remarkable set of cytonemes, and a role for these cytonemes in morphogen-mediated stem cell signalling needs to be addressed in the future (Snyder et al. 2015).

## Wnt signal is distributed on cytonemes

The best characterised cytonemal distributed ligands in vertebrates are members of the Wnt signalling family (Fig. 1). In developmental contexts, cytonemes have been reported to transport Wnt signalling components in various tissues (Holzer et al. 2012; Luz et al. 2014; Stanganello et al. 2015; Sagar et al. 2015; Mattes et al. 2018). Induction of Wnt cytonemes is a remarkable example of the interplay of autocrine and paracrine signalling, and interaction between different Wnt signalling branches. In the sender cell, Wnt signals accumulate at the plasma membranes (Stanganello et al. 2015). There, Wnts binds to the Frizzled receptor and the co-receptor of the planar cell polarity (PCP) pathway Ror2 of the Wnt producing cell (Mattes et al. 2018). Binding of Fzd7/Ror2 to Wnt8a activates the PCP signalling pathway in an autocrine fashion. Activation of Ror2 signalling leads to the induction of Wnt cytonemes in zebrafish (Mattes et al. 2018). The very same Wnt8a clusters, which activate Ror2 signalling, can be found on the resultant cytoneme tip (Fig. 1a, a’). Furthermore, Wnt signal from the PDGFRα positive stroma cells, called telocytes, have been shown to be essential to maintaining the intestinal crypt in mice (Greicius et al. 2018; Shoshkes-Carmel et al. 2018). Blockage of cytoneme formation from these stroma cells by knock-down of Ror2 leads to a loss of Wnt dependent crypt organoids (Mattes et al. 2018). Many cancers require Wnt signalling for proliferation. Wnt signalling is important for regulating tissue homeostasis in the gastrointestinal (GI) tract and aberrant Wnt signalling can lead to GI cancer (Flanagan et al. 2018). Activation of Ror2 signalling in the Wnt producing gastric cancer cells leads to an increase of Wnt-positive cytonemes and enhances Wnt-mediated proliferation in the neighbouring gastric cancer cells. Proliferation of the neighbouring cells can be rescued by reduction of filopodia formation or Wnt signalling (Mattes et al. 2018). These examples illustrate that the Ror2/PCP signalling pathway is a crucial and evolutionary conserved signalling pathway for the control of Wnt dissemination by cytonemes in vertebrates. Mechanistically, Ror2/PCP signalling induces the recruitment of the BAR protein IRSp53, the RhoGTPase Cdc42 and the transducer of Cdc42-dependent actin assembly protein 1 (Toca-1; also known as fnbp1l), all members of the filopodia nucleation machinery (Ho et al. 2004). Outgrowth of a Wnt cytoneme containing the Wnt8a/Fzd7a/Ror2 cluster is observed as a result of F-actin nucleation. The presence of activated Cdc42/N-Wasp in Wnt-containing filopodia that are forming suggests that a dynamic F-actin polymerization takes place continuously during cytonemal outgrowth (Stanganello et al. 2015; Mattes et al. 2018). Subsequently, the Wnt ligand is transported to target cells where it induces the formation of another Wnt receptor–ligand complex—the β-catenin activating Wnt signalosome. The Wnt signalosome consists of an assembly of Fzd-Lrp6 receptors (Bilic et al. 2007) and Dishevelled-Axin heteropolymers (Gammons et al. 2016). The subsequent clathrin-mediated endocytosis of the active signalosome triggers the Wnt/β-catenin transduction cascade (Hagemann et al. 2014). After contact formation, the cytonemes are cut off and a Wnt8a-positive vesicle remains attached to the membrane of the responding cell (Stanganello et al. 2015). In vivo imaging suggested that, after cytonemal contact has been established, Wnt-containing filopodia tips are primarily endocytosed into the recipient cells, which is a prerequisite for signal activation (Hagemann et al. 2014; Gammons et al. 2016; Brunt and Scholpp 2017). In addition to Wnt8a, Wnt2b is also transported on cellular extensions to a Wnt-recipient cell in Xenopus fibroblast cell culture (Holzer et al. 2012). Data from chickens suggest that cytonemes from dermomyotomal cells can also transport the Wnt receptor Fzd7 (Sagar et al. 2015). Therefore, cytonemes are an evolutionary conserved mechanism to mobilise signalling proteins in vertebrate development and tissue homeostasis.

## TNTs transmit signals and organelles between cells

In parallel to the discovery of cytonemes, TNTs have been proposed as another type of contact-dependent cell–cell communication and transport mechanism (Fig. 1d, d’). TNTs were initially discovered by the group of Hans-Hermann Gerdes in 2004 (Rustom et al. 2004). In the following years, many types of cargo could be identified to be transported in or on TNTs. For example, these intercellular bridges enable the mobilization of cargos of various sizes, from signalling proteins to cellular organelles including endosomal vesicles, mitochondria (Rustom et al. 2004; Kadiu and Gendelman 2011; Wang and Gerdes 2012), lipid droplets (Astanina et al. 2015), pathogens (Onfelt et al. 2006; Sowinski et al. 2008), prions (Gousset et al. 2009) and electrical signals (Wang et al. 2010). These versatile cell connections were originally described in primary rat pheochromocytoma (PC12) cultures which build nanotubular bridges that can reach up to several cell diameters to exchange membrane vesicles and organelles (Rustom et al. 2004). Like cytonemes, TNTs are actin-and tubulin-based protrusions, but possess some unique features: once established, TNTs form stable bridges between cells with a seamless membrane connectivity. Electron micrographs suggest a continuous channel that connects the cytoplasm of both cells allowing lateral diffusion of cytoplasm and bi-directional transport of cargo (Rustom et al. 2004). High-resolution structural analysis yields accurate insights into the molecular features of these cellular conduits. Broadly speaking, TNTs can be categorized according to their diameter (Onfelt et al. 2006). Short and thin nanotubes display a diameter of up to a few hundreds of nanometres and a length below 50 µm. Thin TNTs are similar to gap junctions and allow the exchange of smaller cargo such as molecules below 1.2 kDa, including second messengers and small peptides (Ariazi et al. 2017). The second class are longer and thicker TNTs with a diameter over several 100 nm, containing prominent microtubule skeleton and span over hundreds of micrometres (Gerdes et al. 2013). These intracellular bridges can be used to mobilise larger cargo such as organelles or viruses. In the following sections, we will discuss these two groups of TNTs and their involvement in diseases.

## Fine TNTs contain F-actin bundles

In PC12 cells, a shorter and smaller TNT subtype which extend about 20 µm with a diameter of 70–200 nm were characterised. This subtype is mainly based on an F-actin cytoskeleton. Thin intercellular bridges with an average length of 30 µm were also described for a variety of immune cells including human peripheral blood NK cells, macrophages, and B cells. GFP-tagged cell surface class I MHC protein or GPI-conjugated GFP could be transmitted by these stable connections, presenting evidence for a new cell communication mechanism in immunology (Onfelt et al. 2006). Remarkably, thin TNTs also actively transport bacteria along the surface to adjacent macrophages enabling phagocytosis. The TNT connection is thereby merely a guided road, set to a specific destination. Due to the absence of intercellular transport, a fine and simple F-actin-only nanotube seems to save resources within the cell, which allows implementing and rebuilding more TNT connections. In the meantime, various TNT-like structures were described in cell culture. Fine nanotubes are transiently formed between adult human endothelial progenitor cells and neonatal rat cardiomyocytes and were shown to transport GFP. The thin tubes establish a seamless transition between both cells according to the original TNT definition. It is considered that these TNTs influence cell fate decisions in adult progenitor cells (Koyanagi et al. 2005).

## Larger TNTs transport organelles

Tunnelling nanotubes with a diameter over 700 nm were found to transport mitochondria and intracellular vesicles such as endosomes and lysosomes through intercellular connections. For example, UV-stressed PC12 cells—which exhibit an early stage of apoptosis (before the activation of caspase-3)—can be rescued by the transfer of mitochondria via large TNTs from healthy PC12 cells (Wang and Gerdes 2015). In addition to the F-actin bundles, thicker nanotubes also contain microtubules. Microtubule-destabilizing substances such as colchicine and nocodazole prevented intracellular shuttling through these tubes, but not the extracellular surface transport of bacteria. This demonstrates how TNTs can adapt their cytoskeletal assembly and size to match their respective function in delivering a cargo (Onfelt et al. 2006). Cargo with clinical relevance, as shown by wider TNTs, include the transfer of prion aggregates involved in protein-misfolding diseases (PMDs) such as in Alzheimer’s, Huntington’s and Parkinson’s diseases. Outbreak of the disease requires a misfolding of an often unrelated protein into an infectious form coupled with a non-cell autonomous propagation mechanism. Neuronal CAD cells were found to establish membrane bridges that fit the definition of tunnelling nanotubes. These structures were able to transfer infectious PrP^SC^ prions to recipient cells causing a continuous propagation of the disease (Fig. 1b, b’) (Gousset et al. 2009). TNT-mediated transfer also occurred from dendritic cells to neuronal cells or primary neurons, supporting a role for TNT in the spreading of prions from the periphery to the brain. Also in the brain, PrP^SC^ can also mediate by a TNT-like direct cell–cell contact to granule neurons or neuronal cells in co-culture (Victoria and Zurzolo 2017). Prion aggregates were found to be transported in endocytic vesicles such as early endosomes. Similarly, it was shown that a-syn fibrils involved in Parkinson’s disease are transported through TNTs to neighbouring neurons inside lysosomes (Abounit et al. 2016a), underscoring a role for TNTs and lysosomes in the progression of neurodegenerative diseases (Victoria and Zurzolo 2017). Furthermore, a prion-induced stimulation of its own TNT-derived propagation was discovered (Zhu et al. 2015; Abounit et al. 2016b) in which the infectious form of PrP^SC^ as well as Tau, α-synuclein and Htt aggregates increased TNT formation and vesicle transfer. This highlights a mechanism in which the transported cargo plays an active role in its own distribution.

## TNTs in tissue in vivo

In vitro, TNTs were shown to be involved in a multitude of processes, but until recently there was a shortage of data available to emphasise their relevance in vivo. With improvements in fixation methods and live imaging, TNT-like structures can now be described in several tissues. As it is in vitro, the most prominent feature defining TNTs is the continuous and cell-fusing membrane connection. Additionally, the type of cargo transported in TNTs can be used for categorization.

Neural crest cells in chick embryos show TNT-like structures linking two cells by a continuous membrane tether, which is maintained during migration. If broken, it causes a cue for a directional change (Teddy and Kulesa 2004). These bridges actively exchange cytoplasmic material in a bi-directional manner to gain positional information (McKinney et al. 2011). Intercellular bridges were also reported in gastrulating zebrafish embryos that share striking similarities to TNTs (Caneparo et al. 2011). The bridges are different to cytoneme-like protrusions as these bridges are established and then maintained in daughter cells after cell division. Additionally, transfer of cytosolic and membrane-tethered fluorescent proteins was reported in the zebrafish embryos, suggesting that there is a seamless transition from one cell to the other which could mediate cell–cell communication during gastrulation. This continuous membrane tube tethers cells for several hours and can extend up to 350 µm.

Also of note, and as discussed before in case of neurodegenerative diseases (Victoria and Zurzolo 2017), TNTs have been associated with pathogenic roles in diseased tissues. TNTs were discovered between cornea cells in mouse (Seyed-Razavi et al. 2013). Long and F-actin-rich membrane conduits that extend up to 300 µm were observed on donor GFP+ cells establishing contact with resident MHC class II + GFP− host cells in the corneal stroma. An increase of these TNTs considerably enhanced inflammation in the corneas (Chinnery et al. 2008). As the small configuration of these TNTs may impede transfer of larger organelles, their role could lie in the propagation of smaller molecules like MHC-antigen complexes to aid in immune response. These TNTs were formed de novo, extending from the cell body to target cells. TNTs are also used in transmitting viruses. HIV-infected cells spread infection to uninfected cells by TNTs (Okafo et al. 2017). This mechanism helps amplify HIV infection by increasing the probability that small populations of HIV-infected macrophages will spread the infection to a large number of uninfected cells. In cancers, TNTs play a pivotal role in the exchange of information within a tumour. TNTs connect tumour cells of patient-derived malignant pleural mesothelioma to enable a bi-directional transfer of organelles and other cytosolic components (Lou et al. 2012), highlighting a role in mammalian cancer cell pathogenesis and invasion. Similar to TNTs in the cornea, the TNTs of invasive malignant mesothelioma cells are formed de novo to communicate with the surrounding cells. TNTs between stromal mesenchymal cells or endothelial cells and cancer cells were also reported in 3D anchorage-independent spheroids and tumour explants (Pasquier et al. 2013). These findings suggest that TNTs play a role in cell–cell communication in the metastatic niche. In contrast to malignant mesothelioma cells, these nanotubes require initial cell–cell adhesion to form.

Overall, there are a wide range of TNTs observed in vertebrate tissue, however, only a fraction of their functions are understood. Advancements in in vivo imaging have led to an expanding number of discoveries regarding the morphology and function of TNT-like structures.

## TNTs and cytonemes: two of a kind?

TNTs and cytonemes share striking similarities and therefore categorization is sometimes difficult, especially in a complex in vivo setting where there are several types of protrusions which cannot easily be distinguished without further investigation. Studying the composition and morphology of these protrusions is the simplest way to gain insight (Table 1).

**Table 1:** Observations of cytonemes and TNTs in vitro and in vivo

	Cell type and organism	Transmitted signals and cargo	Function	Size: length; diameter	Cytoskeleton element	References
Cytonemes						
In cell culture						
	Xenopus fibroblasts	Wnt2b–EGFP	Wnt secretion	n/a	Actin and microtubules	Holzer et al. (2012)
	Mouse C3H/10T1/2 mesenchymal cells	FGF: FGFR elongation via cytonemes	FGF reception	Up to > 60 µm; n/a	Actin	Koizumi et al. (2012)
	Zebrafish pigment cells	Membrane-associated signal to trigger depolarization	Pigment pattern formation	20–30 µm; n/a	Actin	Hamada et at. (2014)
In tissue						
	Wing disc tracheal cells or peripodial layer of the eye disc	Dpp/Tkv; Spi/EgfR reception	Imaginal disc pattering	Up to > 80 µm; 200 nm	Actin	Sato and Kornberg et al. (2002); Hsiung et al. (2005); Roy et al. (2011)
	Drosophila myoblasts	Wg/Fzd uptake and delta/notch interaction	Relay system for Wg and Notch signals	Up to 25 µm; 200 nm	Actin	Huang and Kornberg (2015)
	Drosophila germ cells	Dpp transport	Maintenance of stem cells	Up to 4 µm	Microtubules	Inaba et al. (2015)
	Basal cytonemes in the Drosophila wing pouch	Hh/patched reception	Imaginal disc pattering	Up to 70 µm; n/a	Actin	Bischoff et al. (2013); Gradilla et al. (2014); González-Méndez et al. (2017)
	Zebrafish neural plate	Wnt transport to Fzd/Lrp6 containing cells	Neuroectoderm AP patterning	Up to 50 µm; <1.5 µm	Actin	Stanganello et al. (2015), Mattes et al. (2018)
	Chick limb bud	Shh ligand caintaining cytonemes connect Cdo and Boc coreceptors cytonemes	Patterning of mesenchymal cells	Up to 150 µm; 200 nm	Actin	Sanders et al. (2013)
Tunnelling nanotubes						
In cell culture						
	Primary rat pheochromocytoma (PC12)	Membrane vesicles, membrane-anchored proteins, and small organelles	Intercellular transfer of cellular components	Thin: 20 µm; 70–200 nm	Actin	Rustom et al. (2004)
	Primary rat pheochromocytoma (PC12)	Mitochondria and intracellular vesicles such as endosomes and lysosomes	Recovery mechanism for stressed cells	Thick: n/a; > 700 nm	Actin and microtubules	Wang and Gerdes (2015)
	Human monocyte-derived macrophages	Mitochondria and intracellular vesicles	Intercellular transfer of cellular components	Thick: n/a; > 700 nm	Actin and microtubules	Onfelt et al. (2006)
	Humanperipheral blood NK cells, macrophages, and B cells	Bacteria ; GFP-tagged cell surface class I MHC protein	Communication mechanism in immunology	10–50 µm; <700 nm	Actin	Onfelt et al. (2006)
	Between adult human endothelial progenitor cells and neonatal rat cardiomyocytes	Mitochondria, soluble GFP	Cell fate decisions in adult progenitor cells	Up to 120 µm; 50–800 nm	Actin	Koyanagi et al. (2005)
	Neuronal CAD cells and between astrocytes to granule neurons	PrP^SC^ prions	Propagation of infectious particles	Up to 80 µm; 180–800 nm	Actin	Gousset et al. (2009); Zhu et al. (2015)
	NRK cells, HEK293, HUVEC, and NCC	Electrical signals	Electrical coupling via gap junctions	Up to 70 µm; 50–200 nm	Actin	Wang et al. (2010)
In tissue						
	Neural Crest cells in chick embryos	Cytoplasmic material; cytosolic and membrane-tethered fluorescent proteins	Delivering positional information during migration	Up to 100 µm; 0.5–2 µm	N/a	Teddy and Kulesa (2004); McKinney et al. (2011)
	Gastrulating zebrafish embryo cells	Cytosolic and membrane-tethered fluorescent proteins	Cell–cell communication during gastrulation	Up to 350 µm; <1 µm	Actin; tubulin only in proximal part	Caneparo et al. (2011)
	Myeloid cells in the mouse cornea	Smaller molecules such as MHC and/or MHC-antigen complexes	Immunological response	> 300 µm; 200–300 nm	Actin; n/a	Chinnery et al. (2008); Seyed-Razavi et al. (2013)
	Patient-derived malignant pleural mesothelioma	Organelles and other cytosolic components	Role in pathogenesis and invasion	Up to 100–200 µm; n/a	Actin; n/a	Lou et al. (2012)

For instance, two distinct types of cellular bridges were spotted during neural tube closure in cultured mouse embryos (Pyrgaki et al. 2010). Non-neural ectodermal cells extend protrusions across and between the gap of the closing fold during neurulation. One extension was said to feature cytoneme characteristics due to its mobile and flexible attributes and was speculated to promote the formation of stable intermediate closure points. The more robust protrusions can be described as nanotube-like structures. The TNT-like structures are more rigid and physically bridge cells between the closing folds. Of note, this was one of the first observations describing TNTs in mammalian tissue, however, their function and cargo are still unknown.

Focussing on only the physical properties of the protrusions is insufficient for an explicit assignment to a category; expanding the analysis to include a description of the cargo transported can also separate TNTs from cytonemes. Membrane connections in T-cells feature striking similarities to TNTs at first glance as they appear to firmly bridge cells for a transfer and presentation of viral HIV-1 particles (Sowinski et al. 2008). However, as the characteristics of the protrusion did not match all defined criteria, it was labelled TNT-like. One reason was the absence of membrane continuity. A more striking disparity might be the mode of how cell–cell contact is established. The cell–cell contact was mediated by the receptor CD4 on the protrusion tip and the viral protein Env, which resembles cytoneme-like characteristics. Instead of a direct transfer through the membrane channel, ligand–receptor interaction leads to a subsequent phagocytosis of the TNT tip (Sowinski et al. 2008). As the receptor–ligand interaction resembles a signalling process, it could be speculated whether this protrusion illustrates a cytoneme rather than a TNT.

The broad amount of diverse functions and modes of delivery is also a factor to compare. As cytonemes were described as signalling filopodia in cell–cell communication, the signalling purpose stands out as a vital criterion. Integral to signalling activity, the transfer of ligands and receptor–ligand interactions—often followed by subsequent pathway activation—is the second criterion. The scope of application is much broader for TNTs. TNTs are able to transfer various molecular, electrical or mechanical signals, but the allocation relied mainly on the physical composition of the protrusion itself. In addition, the directionality of cargo transport displays diversities as well. While TNTs were shown to transport in a bi-directional fashion (Rustom et al. 2004; Teddy and Kulesa 2004; Lou et al. 2012), permitted by the open-ended protrusion, cytonemes operate as a one-way road (Kornberg and Roy 2014; Stanganello and Scholpp 2016). In fact, the separation in producing and signal receiving tissue is a notable aspect of cytoneme biology.

## Formation of TNTs versus cytonemes

In relation to the formation of these specialized cell protrusions, TNTs again exhibit greater heterogeneity in comparison to cytonemes. Cytonemes share striking similarities with classic filopodia in both their molecular composition as well as in the formation and subsequent elongation of these F-actin-driven protrusions. The formation occurs de novo and involves the stimulation of an N-WASP nucleation complex activated by RhoGTPases such as Cdc42, Rac1 or RhoD (Ho et al. 2004; Faix and Rottner 2006; Stanganello et al. 2015). Elongation of cytonemes follows the rules of filopodia extension by requiring actin polymerization stimulators such as the Arp2/3 and Ena/VASP complex as well as F-actin bundling mediated by fascin1 (FSCN1).

In contrast, TNTs were demonstrated to result from two completely diverse events but seem to be not mutually exclusive as they can be observed in the same cell models. One involves similar procedures as in filopodia formation, by sprouting and extension of filopodia after initiation of the actin nucleation utilizing the Cdc42 machinery (Faix and Rottner 2006). This was illustrated in various cell lines (Rustom et al. 2004; Gousset et al. 2009) as well as in vivo in cornea cells of mice (Seyed-Razavi et al. 2013). The molecular events following filopodia anchoring after cell–cell contact are unclear, and a mechanism to convert a fragile filopodia structure into a stable membrane tether is missing. Furthermore, TNTs exhibit membrane continuity that is achieved by the merge of the filopodia tip to the target cell membrane in which SNARE and viral fusion proteins were shown to provide the energy for the fusion process (Martens and McMahon 2008).

A further method for TNT formation was termed the cell dislodgment mechanism because two tightly attached cells, typically after an event of cell division, migrate apart while retaining a membrane tether (Onfelt et al. 2006; Caneparo et al. 2011). Historically, the linkage of daughter cells has precedent with the ring canals that connect germline nurse cells in Drosophila. These connections persist between imaginal disc cells for some period of time after cell division. Resulting from the tight connection, membrane fusion is already present before the extension process starts, therefore the subsequent migration apart might be purely an expansion and maintenance of the initial cell–cell connection that enables a steady way for cell–cell communication and cargo transfer.

However, the molecular mechanism for TNT formation is still far from understood, especially as different formation models and the disparities of cell behaviour in between cell lines impede our understanding. The methods share the similar actin-associated machinery, however, there is evidence showing an opposed influence of key regulatory complexes for either filopodia or TNT formation (Gousset et al. 2013; Delage et al. 2016). A recent study demonstrated an opposing molecular mechanism for TNTs and filopodia formation by different functions of the shared CDC42/IRSp53/VASP network in mouse neuronal CAD cells. CDC42, IRSp53 and VASP negatively regulated TNT formation by reducing the number of TNTs connected cells and the vesicle transfer (Delage et al. 2016). Interestingly, CDC42/IRSp53/VASP act as positive stimulators for filopodia in the same cells. This discrepancy was also shown for other actin regulators such as Fascin32 and EGFR pathway substrate 8 (Eps8) (Gousset et al. 2013).

These findings provide a novel insight about unique formation mechanisms of TNTs and to differentiate them from signalling filopodia-the cytonemes. Elsewhere, the same molecular machinery was also found in other biological contexts to conduct a positive influence on TNTs as well as on filopodia formation (Arkwright et al. 2010; Schiller et al. 2013). To address this molecular heterogeneity, more comparative work is needed.

## Summary

In this review, we discussed common features of cytonemes and TNTs. TNTs and cytonemes transfer information between cells in a tissue and organs with often a common functionality. Besides functional commonalities, these structures display a great number of unique features especially regarding the process of formation and the type of cargo transported. In the light of new data, the hypothesis that TNTs could arise from a subset of filopodia seems to be unlikely. It is probably rather useful to see outgrowing filopodia and TNT precursors as different structures from the beginning. A difference—which is more serious—is a conceptual one: the essence of cytonemes is the regulated exchange of signals. The delivery of signals by cytonemes is quantitatively, temporally, and spatially precise, irrespective of the distance between the communicating cells. TNTs are the opposite—they resemble “open channels” with shared cytoplasm—so the regulation of cargo cannot be as tightly controlled as for cytonemes. Regardless of these differences, both structures fulfil important functions in the exchange of information within a tissue and are vital parts of an information network in tissues. A thorough functional and structural characterisation of cytonemes and TNTs and their interactions in contact-based signalling is fundamental and calls for further studies at the molecular, cellular and tissue level.
